# Genetic architecture of kernel composition in global sorghum germplasm

**DOI:** 10.1186/s12864-016-3403-x

**Published:** 2017-01-05

**Authors:** Davina H. Rhodes, Leo Hoffmann, William L. Rooney, Thomas J. Herald, Scott Bean, Richard Boyles, Zachary W. Brenton, Stephen Kresovich

**Affiliations:** 1 Center for Grain and Animal Health Research, USDA-ARS, 1515 College Ave, Manhattan, Kansas 66502 USA; 2 Department of Soil & Crop Sciences, Texas A&M University, College Station, TX 77843 USA; 3 Institute of Translational Genomics, Clemson University, Clemson, SC 29634 USA

**Keywords:** Sorghum, GWAS, QTL, Natural variation, Grain composition, Cereal crop, Nutrition

## Abstract

**Background:**

Sorghum [*Sorghum bicolor* (L.) Moench] is an important cereal crop for dryland areas in the United States and for small-holder farmers in Africa. Natural variation of sorghum grain composition (protein, fat, and starch) between accessions can be used for crop improvement, but the genetic controls are still unresolved. The goals of this study were to quantify natural variation of sorghum grain composition and to identify single-nucleotide polymorphisms (SNPs) associated with variation in grain composition concentrations.

**Results:**

In this study, we quantified protein, fat, and starch in a global sorghum diversity panel using near-infrared spectroscopy (NIRS). Protein content ranged from 8.1 to 18.8%, fat content ranged from 1.0 to 4.3%, and starch content ranged from 61.7 to 71.1%. Durra and bicolor-durra sorghum from Ethiopia and India had the highest protein and fat and the lowest starch content, while kafir sorghum from USA, India, and South Africa had the lowest protein and the highest starch content. Genome-wide association studies (GWAS) identified quantitative trait loci (QTL) for sorghum protein, fat, and starch. Previously published RNAseq data was used to identify candidate genes within a GWAS QTL region. A putative alpha-amylase 3 gene, which has previously been shown to be associated with grain composition traits, was identified as a strong candidate for protein and fat variation.

**Conclusions:**

We identified promising sources of genetic material for manipulation of grain composition traits, and several loci and candidate genes that may control sorghum grain composition. This survey of grain composition in sorghum germplasm and identification of protein, fat, and starch QTL contributes to our understanding of the genetic basis of natural variation in sorghum grain nutritional traits.

**Electronic supplementary material:**

The online version of this article (doi:10.1186/s12864-016-3403-x) contains supplementary material, which is available to authorized users.

## Background

Chronic hunger can be alleviated by improving the nutrition of staple cereal crops, which provide the majority of nutrients to the world’s population [1]. Grain composition varies within and among cereal crops, but, generally, grain contains 79–83% starch, 7–14% protein and 1–7% fat. Crop yields in the arid and semi-arid regions of the world are challenged by low precipitation, leaving populations in these regions particularly vulnerable to chronic hunger and malnutrition. Sorghum is a cereal crop that is well adapted to regions of low precipitation, and thus, has become a staple crop that feeds millions of people in sub-Saharan Africa [2], where the highest prevalence of undernourishment in the world is found [3]. Understanding the natural variation of protein, fat, and starch, and identifying QTL associated with their natural variation in sorghum grain can help improve its nutritional quality through crop improvement programs and marker-assisted selection.

Until the seed is self-sustaining, protein, fat, and starch stores are used to support the developing seedling. Since these nutrient stores are also critical components of the human diet, many researchers have focused on improving the nutrient composition of seeds [4]. For instance, the Illinois long-term selection experiment, which began in 1896, has increased the oil and protein content of maize inbred lines to 20 and 27%, respectively, compared to ~6 and ~12%, in an average maize line [5–8]. The chemical composition of grain is controlled by complex regulations that takes place during the seed filling stage of seed maturation, when protein, fat, and starch storage compounds accumulate [9–11]. Key insights into the genetic controls of grain composition have been discovered through several rice and maize mutations with altered grain composition, including *opaque-2* and *floury-2*, which affect protein content [12–15]; *linoleic1* and *fad2,* which affect fat content [16–18]; and *shrunken1* and *amylose extender1*, which affect starch content [19–21]. Sorghum mutations have also contributed to our knowledge of the genetic controls of grain composition. These mutations include *waxy*, which has little to no amylose, increased protein, and improved starch digestibility [22–24]; *sugary*, which has increased sucrose content [25, 26]; and *high-lysine*, which has increased lysine content and protein digestibility [27].

GWAS have identified allelic polymorphisms for important agronomic traits in cereal crops [28–32], including alleles responsible for variation in grain composition of rice [30, 32], maize [33–36], and barley [37, 38]. Linkage and association studies have identified several loci controlling sorghum grain composition [39–43], and the identification of the gene underlying the *waxy* mutation has been fine mapped to 1.8 Mb on chromosome 10 [44], but more work needs to be done to precisely identify genes responsible for natural variation of grain composition. GWAS for sorghum grain composition have identified QTL for polyphenol [45] and mineral traits [46], but no GWAS have been conducted for protein, fat, and starch composition.

Surveying the natural variation of grain composition in the sorghum germplasm and finding loci underlying the variation can aid efforts to improve the nutritional value of sorghum. New sources of genetic variation can be used for crop improvement, especially in developing countries where technologies that exist for improving the nutritional value of grain, such as commercial fortification, are not accessible or affordable [47–49]. The goals of this study were to quantify natural variation of sorghum grain protein, fat, and starch and to identify associated SNPs. Here, we characterize the natural variation of sorghum grain composition in a global sorghum diversity panel and use GWAS to identify allelic variation underlying variation in grain composition.

## Methods

### Plant materials

We grew 390 sorghum accessions from the Sorghum Association Panel (SAP) [50]. The panel includes important breeding lines from the United States and traditional varieties from all five major races (bicolor, guinea, caudatum, kafir, and durra) and 10 intermediate races (all combinations of the major races) [51]. Seeds were originally obtained from the U.S. National Plant Germplasm System’s Germplasm Resources Information Network (GRIN) [52] and planted in late April to May 2012, 2013, and 2014 at Clemson University Pee Dee Research and Education Center in Florence, SC. The field design has been described previously [28]. Briefly, a two-fold replicated complete randomized block design was used. Panicles from each plot were collected at physiological maturity (black layer), which occurs once grain filling is complete. Due to differences in maturity among these accessions, harvest occurred between September and October. Once harvested, panicles were air dried in a greenhouse and mechanically threshed. In the following analyses we consider 265 accessions for which we obtained replicated data in all 3 years.

### Phenotyping

Protein, fat, and starch content were measured using NIRS at Texas A&M University’s sorghum breeding and genetics lab. Twenty grams of cleaned whole grain were scanned with a FOSS XDS spectrometer (FOSS North America, Eden Prairie, MN, USA). The NIR reflectance spectra were recorded using the ISIscan software (Version 3.10.05933) and converted to estimates using in-house developed models for protein, fat, and starch concentrations (expressed as a percentage of dry weight). The total grain weight in grams of 100 grains per accession was recorded. Analysis was conducted on the mean trait values across years.

### Genomic analysis

Genotypes were available for all of the accessions [53]. GWAS was carried out on 404,627 SNP markers, using the statistical genetics package Genome Association and Prediction Integrated Tool (GAPIT) [54]. SNPs with a minor allele frequency (MAF) less than 0.05 and with more than 20% missing data were removed from analysis, leaving 141,310 SNPs. A unified mixed linear model (MLM) [55] with kinship, which accounts for relatedness among the accessions in the panel, was performed [56]. Multiple testing was controlled with a false discovery rate (FDR) of 5% using the Benjamini and Hochberg procedure [57] implemented in GAPIT. Narrow-sense heritability was calculated in GAPIT using a compressed mixed linear model that uses the genetic marker-based kinship matrix to estimate additive genetic effects [54]. Linkage disequilibrium (LD) was calculated using Tassel 5.2 [58]. Prior to conducting GWAS, we carried out an extensive literature search to identify potential candidate genes, and used *Sorghum bicolor* genome v1.4 from Phytozome [59] to compile a list of previously identified candidate genes associated with grain composition [35, 36, 60], as well as genes known to be involved in grain maturation and grain filling [9, 11, 61, 62] in Arabidopsis, rice, and maize, resulting in a list of 430 *a priori* gene candidates (Additional file 1). To analyze population structure of the SAP, we used previously published genetic groupings that were determined through Bayesian hierarchical clustering analysis [29]. Five genetic groupings were used and we designated them group A through E (Additional file 2).

### Expression data

To identify candidate genes within a significantly associated region, we used RNAseq data that was generated as a community resource for transcriptomic analyses [63]. Genes in a QTL region that were expressed during grain maturation were considered strong candidates. Expression levels were reported in fragments per kilobase of exon per million reads mapped (FPKM). We used the definitions of Davidson et al [63], as follows: FPKM ≤ 1 = “not expressed”; FPKM ≤ 4 = “low-expressed”; FPKM between 4 and 24 = “intermediate-expressed”; and FPKM ≥ 24 = “high-expressed”.

## Results

### Phenotypic variation and heritability of sorghum grain composition

**Fig. 1:**
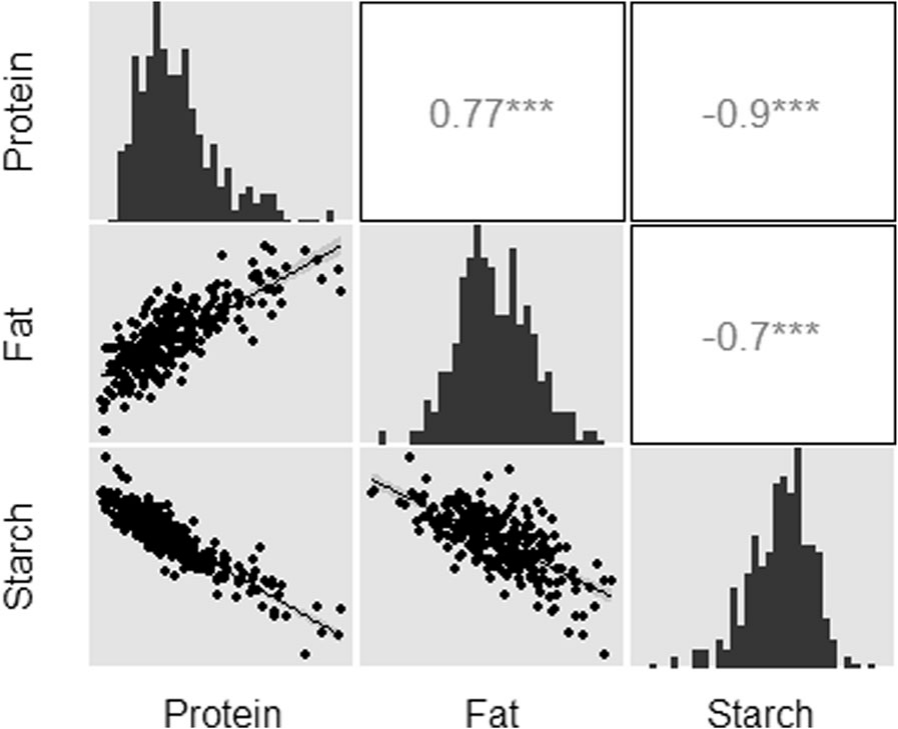
Relationship of grain composition traits in a sorghum germplasm collection averaged over 3 years. The center diagonal presents histograms of each trait. The scatter plots with regression lines show the relationships between the traits. (*n* = 265)

Overall, grain composition was similar across years, with protein, fat, and starch all having a strong correlation across years. Protein was the most consistent at 73–82% correlation between the 3 years, whereas fat (57–69%) and starch (51–65%) had slightly lower year to year correlations (Additional file 3). Similarly, protein had the highest narrow-sense heritability (*H* ^*2*^ = 0.90), followed by fat (*H* ^*2*^ = 0.85) and starch (*H* ^*2*^ = 0.80). Next, we investigated the range of sorghum grain protein, fat, and starch content and their covariation with each other using the mean of the 3 years (Additional file 4). The germplasm showed a wide range of diversity in grain composition. Protein content ranged from 8.3 to 18.8%, fat content ranged from 1.0 to 4.4%, and starch content ranged from 61.7 to 70.8% (Fig. 1). Pearson’s correlations were calculated between protein, fat, and starch (Fig. 1). There was a strong negative correlation between starch and both protein (*r* = -0.90, *p* < 10^−16^) and fat (*r* = -0.70, *p* < 10^−16^), and a strong positive correlation between protein and fat (*r* = 0.77, *p* < 10^−16^). When grain composition concentrations are expressed as percentage by total seed weight, an increase in one component decreases the percentage of other components. Therefore, the percent concentration was multiplied by the seed weight of each accession to get absolute estimates of the mass of each constituent per grain, and Pearson’s correlations were recalculated. Using these estimates, there was a positive correlation between starch and both protein (*r* = 0.66, *p* < 10^−16^) and fat (*r* = 0.56, *p* < 10^−16^), and a strong positive correlation between protein and fat (*r* = 0.85, *p* < 10^−16^). In contrast to correlations when using the percent concentration, the positive correlations between the mass of the traits reflect that total amounts of protein, fat, and starch increase with increases in total seed weight.

Next we investigated grain protein, fat, and starch covariation with factors that could reduce their biological availability for human consumption. Since the digestibility of protein and starch can be decreased by proanthocyanidins, and possibly other polyphenols [64], it is useful to know if there is a pattern of covariation between grain composition traits and polyphenol content. To this end, we used previously generated polyphenol data that was measured in the same samples as the current study [45] to calculate Pearson’s correlations with starch, protein, and fat concentrations (Additional file 5). Starch was negatively correlated with total polyphenols (*r* = -0.34, *p* < 10^−9^), proanthocyanidins (*r* = -0.29, *p* < 10^−6^), and 3-deoxyanthocyanidins (*r* = -0.29, *p* < 10^−6^); protein was positively correlated with total polyphenols (*r* = 0.42, *p* < 10^−12^), proanthocyanidins (*r* = 0.34, *p* < 10^−8^), and 3-deoxyanthocyanidins (*r* = 0.28, 10^−6^); while fat was only positively correlated with total polyphenols (*r* = 0.25, *p* < 10^−5^) and proanthocyanidins (*r* = 0.20, *p* = 0.001).

### Population structure of grain composition traits

Knowledge of variation in grain composition across genetically similar sorghum groups can be applied to germplasm utilization. Using five genetic groupings, population differences in grain composition were determined (Table 1). Group A consisted of 46 accessions that were primarily durras and bicolor-durras from Ethiopia and India; group C consisted of 55 accessions that were primarily kafirs from USA, India, and South Africa; group D consisted of 23 accessions that were primarily caudatums and guineas from Nigeria and USA; and group E consisted of 117 accessions that were primarily caudatums from Sudan and USA. Group B consisted of only 2 accessions, so was not included in analysis. Group A had the highest protein (12.6%) and fat (3.0%) and the lowest starch (66.1%), while group C had the lowest protein (10.9%) and the highest starch (67.6%).

**Table 1:** Population structure of grain composition traits in a global sorghum germplasm collection^a^

Genetic group	Number of accessions	Protein (%)^c^	Fat (%)^c^	Starch (%)^c^	Predominant race	Predominant country
A	46	12.6 ± 2.3^a^	3.0 ± 0.6^a^	66.1 ± 1.6^a^	durra, bicolor-durra	Ethiopia, India
B^b^	2	13.1 ± 3.4	3.5 ± 1.4	65.9 ± 1.0	bicolor-guinea	Ethiopia
C	55	10.9 ± 2.0^b^	2.7 ± 0.6^a,b^	67.6 ± 1.2^b^	kafir	USA, India, S. Africa
D	23	11.6 ± 1.8^a^	3.0 ± 0.5^a^	67.2 ± 1.3^b^	caudatum, guinea	Nigeria, USA
E	117	11.2 ± 1.8^b^	2.6 ± 0.6^b^	67.1 ± 1.2^b^	caudatum	Sudan, USA, Ethiopia, Uganda

^a^Data represent mean grain composition concentrations across 3 years

^b^Genetic grouping B was not considered in analysis due to the small sample size

^c^Values in the same column with different letters are significantly different from each other, based on a post hoc Tukey HSD test

### Genome wide association study

**Fig. 2:**
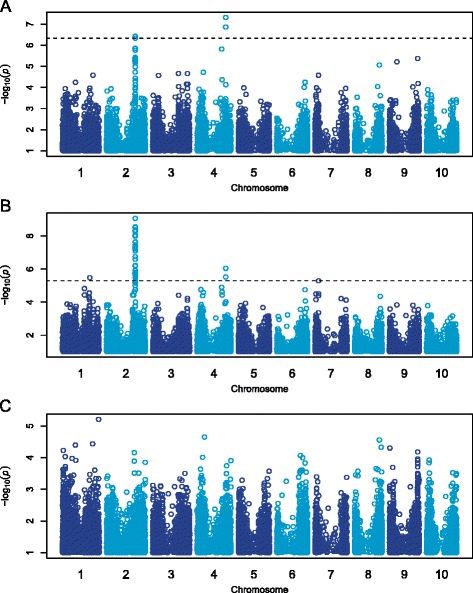
GWAS for protein, fat, and starch content in sorghum grain. Manhattan plots of association results from a MLM analysis using 265 accessions. Each point represents a SNP, with the -log10 p-values plotted against the position on each chromosome. SNPs with MAF < 0.05 were removed. The horizontal dashed line represents the genome-wide significance threshold at 5% FDR. **a** protein; **b** fat; **c** starch

To investigate the genetic basis of natural variation of protein, fat, and starch in sorghum grain, we conducted a GWAS using 265 accessions from the diverse association panel. Control experiments to support the validity of the GWAS results are described in the supplemental material (Additional file 6). Using the estimated mass of protein, fat, and starch, the MLM identified 4, 41, and 0 significant SNPs, respectively, at a genome-wide FDR of 5% and MAF ≥ 0.05 (Fig. 2a-c; Additional file 7). For both protein and fat, there was an association peak on chromosome 4 between 57.6 and 57.7 Mb and on chromosome 2 between 57.6 and 57.7 Mb. All of the significant SNPs on chromosome 2 (with the exception of S2_57592740) are in partial to strong LD with each other (*r* ^*2*^ = 0.5–1.0). Most of the SNPs in the chromosome 2 peak are in partial LD (*r* ^*2*^ = 0.5) with an *a priori* candidate gene that is a putative homolog of alpha-amylase 3 (*AMY3*, Sb02g023790; 57,701,214–57,703,517 bp). This gene is expressed in the day 10 seeds (2.1 FPKM) and in the endosperm (5.8 FPKM; Additional file 8).

**Fig. 3:**
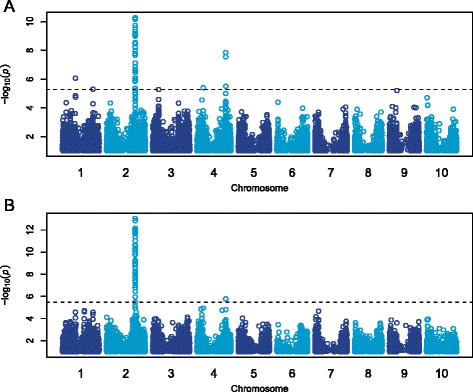
Residuals GWAS for protein and fat content in sorghum grain. Manhattan plots of association results from a MLM analysis using 265 accessions. Each point represents a SNP, with the -log10 p-values plotted against the position on each chromosome. SNPs with MAF < 0.05 were removed. The horizontal dashed line represents the genome-wide significance threshold at 5% FDR. **a** protein; **b** fat

Since starch makes up the majority of the grain, it is possible that some variation in protein and fat content are driven by variation in starch content. We hypothesized that natural variation in starch pathways might be affecting protein and fat content in the grain due to a limited pool of carbon. To determine if starch could be influencing the values, we ran two linear models in which we fit either protein or fat as the dependent variable and starch as the independent variable, using their estimated mass. If we assume that patterns in protein and fat are driven by starch, then starch could account for a significant proportion of the variance—45% of the variance in protein (*p* < 10^−16^) and 32% of the variance in fat (*p* < 10^−16^)—but there is a large portion of variance still unexplained. Therefore, we conducted GWAS on the residuals (the amount of variation in protein and fat that could not be explained by starch) from the linear models to determine if accounting for this variation allowed for more accurate mapping results. The GWAS for protein and fat residuals identified 40 and 45 significant SNPs, respectively, at the FDR adjusted significance threshold, all within the peak on chromosome 2 at ~57.6 Mb and the peak on chromosome 4 at ~57.6 Mb (Fig. 3a-b; Additional file 7).

## Discussion

### QTL for kernel composition

GWAS for protein and fat in the sorghum global diversity panel identified two major peaks in common, one on chromosome 2 at 57.7 Mb and the other on chromosome 4 at 57.7 Mb. The peak on chromosome 2 at 57.7 Mb remained when GWAS was performed on the individual biological replicates in each year (Additional file 1). The peak is near a grain fat QTL from a sorghum linkage study that used a biparental population (Rio X BTx623) grown in Texas [41]. The previously identified grain fat QTL on chromosome 2 is near the genetic marker txp298 at ~57 Mb [65]. A promising *a priori* candidate near this peak is an *AMY3* homolog. AMY3 is an alpha-amylase debranching enzyme that hydrolyzes the glucosidic bonds that make up starch. *AMY1* was previously identified as a candidate gene in a maize grain composition GWAS study [35]. A recent study using AMY3 overexpression lines found that the increased levels of AMY3 did not significantly affect starch content, but fat content was increased in the mature endosperm where starch had been partially degraded [66]. The authors suggested that starch degradation during grain maturation led to the release of sucrose that was then shunted into the Kennedy pathway for fat synthesis.

### Improvement of sorghum grain composition for human nutrition

The range of protein, fat, and starch content found in our diverse association panel may be useful for sorghum improvement. Genetic group A, consisting of durra and bicolor-durra sorghums had significantly higher mean protein levels than the other groups, and are promising sources of genetic material for high protein sorghums. Durra sorghums are genetically similar to bicolor sorghums [29], which is the least derived race (i.e., retains most similarity to wild ancestors among the races), and high protein varieties may have been inadvertently counter-selected during cereal domestication when high starch varieties were selected. It may be that human selection for different food uses influenced the patterns of grain composition distribution among genetic groups (e.g., thick porridge from one region requires a certain grain composition, whereas flat bread from another region requires a different grain composition). It could also be that adaptation to environmental factors is driving some of the grain composition differences between genetic groups. Evidence of this adaptation was recently found for tannins in sorghum grain, when a variant of the *Tannin1* gene, which controls the presence of tannins in the grain, was found to be correlated with several bioclimatic factors [53].

This study provides genetic trait association loci that can be explored further for their potential use in molecular breeding to modify the composition of grain sorghum. The high heritability of each trait suggests the genetic contribution to variation is strong. However, a GWAS with the SAP grown in Kansas (Additional file 6) did not identify the same large association peaks identified in the GWAS in the current study, suggesting a genotype-by-environment interaction. Several previous studies have found grain composition variation between environments, indicating that at least some genes may only be influential in a particular environment [67]. For example, in the biparental population (Rio X BTx623) grown in Texas genotype-by-environment effects explained a significant proportion of phenotypic variability in grain protein, fat, and starch [41]. This suggests that a more systematic investigation of genotype-by-environment interaction on grain composition may be needed to guide breeding efforts.

Genetic correlations among traits can complicate improvement of any single trait. The shared QTL for protein and fat in sorghum grain raises the question of whether protein and fat levels can be selected independently. Several other studies have found strong correlations and shared QTLs between protein, fat, and starch, as well as between these traits and grain yield [6, 35, 39, 41, 43, 68–71]. Shared genetic controls or developmental mechanisms of the grain components may be the cause of the correlations, however, some of the correlations may be due to evolutionary correlations rather than a shared genetic or developmental basis. Further studies to identify genes that control each grain composition trait could be useful. Since biparental mapping populations break up the evolutionary correlations present in association panels, they can be used to determine if the associations are due to a shared genetic basis or to evolutionary history.

## Conclusions

Promising sources of genetic material for manipulation of grain composition traits have been identified, as well as several loci and candidate genes that may control sorghum grain composition. The starch GWAS did not identify any significant SNP associations, implying that, given the high heritability of starch and the lack of significant QTL, starch variation is likely controlled by many small effect genes. Biparental mapping or nested association mapping may be helpful in identifying starch gene candidates. Identification of a highly significant peak on chromosome 2 associated with protein and fat provides a good starting point for marker-assisted breeding of sorghum grain composition traits. This survey of grain composition in sorghum germplasm and identification of QTL significantly associated with protein and fat contributes to our understanding of the genetic basis of natural variation in sorghum grain composition.

## Additional files

Additional file 1:
*a priori* gene candidate list. (XLSX 49 kb)
Additional file 2: Five genetic groupings of sorghum association panel. (XLSX 18 kb)
Additional file 3: Relationship of three separate years of grain composition traits in a sorghum germplasm collection. (PDF 138 kb)
Additional file 4: Protein, fat, and starch content of sorghum accessions. (XLSX 21 kb)
Additional file 5: Relationship within and between grain composition traits and polyphenol content. (PDF 90 kb)
Additional file 6:
**Table S6.1**﻿. ﻿Correlations between traits in Kansas panel^a^ and South Carolina panel. **Figure S6.1.** QQ plots. **Figure S6.2.** GWAS for protein, fat, and starch content in sorghum grain grown in 2012. **Figure S6.3.** GWAS for protein, fat, and starch content in sorghum grain grown in 2013. **Figure S6.4** GWAS for protein, fat, and starch content in sorghum grain grown in 2014. **Figure S6.5.** GWAS for protein, fat, and starch content in sorghum grain grown in Kansas. **Figure S6.6.** GWAS for flowering time in sorghum grain. (PDF 1087 kb)
Additional file 7: Significant SNPs associated with protein, fat, and starch. (XLSX 29 kb)
Additional file 8: Expression data for genes under major peaks. (XLSX 25 kb)

## Abbreviations

*AMY3*: Alpha-amylase 3
FPKM: Fragments Per Kilobase of exon per Million fragments mapped
GRIN: Germplasm Resources Information Network
GWAS: Genome-wide association study
Mb: Mega basepairs
MLM: Mixed linear model
NIRS: Near-infrared spectroscopy
QTL: Quantitative trait loci
SNP: Single-nucleotide polymorphism
