# Supplementary material for: Genetic architecture of kernel composition in global sorghum germplasm
Source: BMC Genomics. 2017 Jan 5;18:15. doi: 10.1186/s12864-016-3403-x (PMC5217548; doi:10.1186/s12864-016-3403-x)
Supplement: Additional file 3: — Relationship of three separate years of grain composition traits in a sorghum germplasm collection. (PDF 138 kb) [file 12864_2016_3403_MOESM3_ESM.pdf]

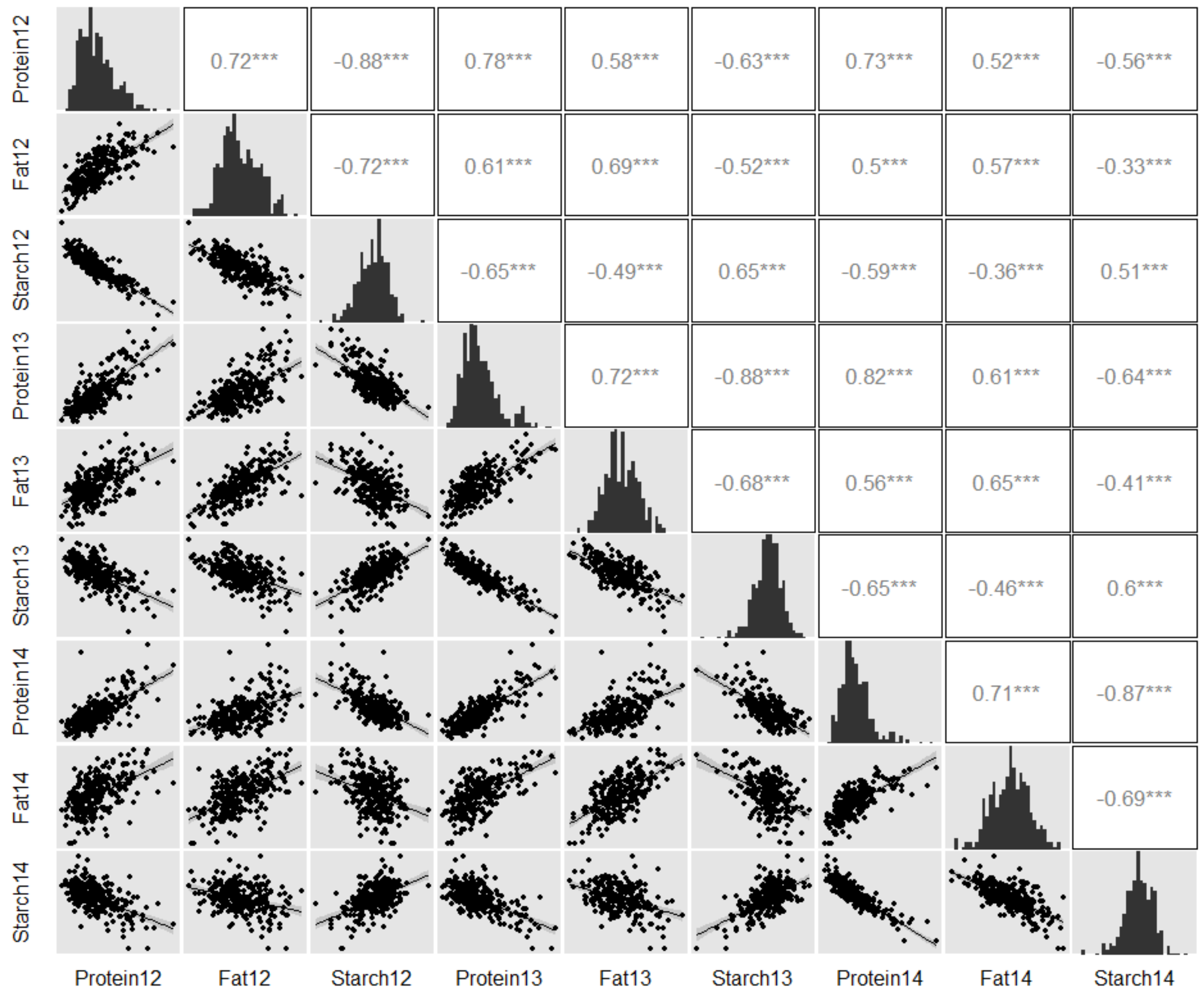

Additional file 3. Relationship of three separate years of grain composition traits in a sorghum germplasm collection. The center diagonal presents histograms of each trait. The scatter plots with regression lines show the relationships between the traits. (n = 265)
