# Supplementary material for: Genetic architecture of kernel composition in global sorghum germplasm
Source: BMC Genomics. 2017 Jan 5;18:15. doi: 10.1186/s12864-016-3403-x (PMC5217548; doi:10.1186/s12864-016-3403-x)
Supplement: Additional file 5: — Relationship within and between grain composition traits and polyphenol content. (PDF 90 kb) [file 12864_2016_3403_MOESM5_ESM.pdf]

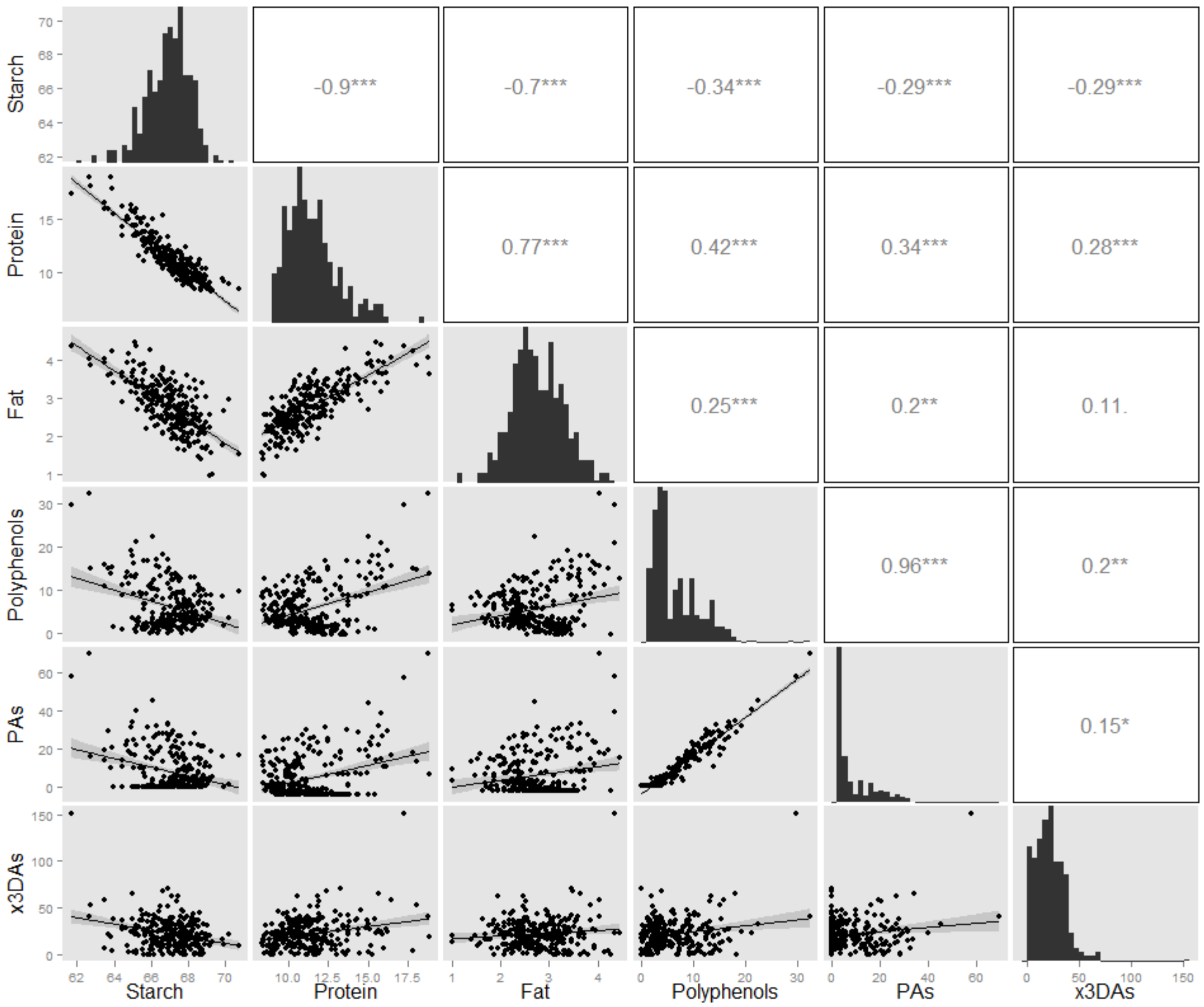

Additional file 5. Relationship within and between grain composition traits and polyphenol content. The center diagonal presents histograms of each trait. The scatter plots with regression lines show the relationships between the traits. (*n* = 265)
