# Supplementary material for: Genetic architecture of kernel composition in global sorghum germplasm
Source: BMC Genomics. 2017 Jan 5;18:15. doi: 10.1186/s12864-016-3403-x (PMC5217548; doi:10.1186/s12864-016-3403-x)
Supplement: Additional file 6: — Table S6.1﻿. ﻿Correlations between traits in Kansas panela and South Carolina panel. Figure S6.1. QQ plots. Figure S6.2. GWAS for protein, fat, and starch content in sorghum grain grown in 2012. Figure S6.3. GWAS for protein, fat, and starch content in sorghum grain grown in 2013. Figure S6.4 GWAS for protein, fat, and starch content in sorghum grain grown in 2014. Figure S6.5. GWAS for protein, fat, and starch content in sorghum grain grown in Kansas. Figure S6.6. GWAS for flowering time in sorghum grain. (PDF 1087 kb) [file 12864_2016_3403_MOESM6_ESM.pdf]

Control Analysis on GWAS QTL

Quantile-quantile (QQ) plots were generated in GAPIT and indicate that false-positive SNP-trait associations due to population structure and kinship were well controlled in the mixed model (Figure 6.1A-C). As a control analysis we used the replicate samples from our dataset, which were grown in a two-fold block design, to conduct GWAS separately on data from each block in each of the three growing seasons. GWAS identified the same large association peak on chromosome 2 at 57.7 Mb when run separately on each block and in each year (Figure 6.2-6.4). To test if the GWAS QTL are stable across environments, we conducted a GWAS using phenotype data from a sorghum panel grown in Kansas in 2007 and 2008 that primarily consisted of the Sorghum Association Panel [1]. Significant correlations were found between the Kansas panel and each year of the South Carolina panel for protein and starch (Table 6.1). No SNPs reached the FDR adjusted significance threshold and there were no obvious association peaks (Figure 6.5). Since phenotypic covariates are a potential source of misleading associations [2], we wanted to know if maturity differences in our diverse panel influenced the grain quality GWAS results. Maturity differences across the panel can potentially lead to grain composition differences, causing true associations with maturity rather than grain composition. If maturity loci were affecting grain quality, then we would expect grain quality QTL to colocalize with maturity loci. With this in mind, we conducted a GWAS using flowering time data from the 2012 samples, which was determined by the number of days from planting until the start of anthesis. The major peak in the GWAS mapped to the previously identified maturity locus, *ma1* (chromosome 6, ~42 Mb) [3,4] and, importantly, did not map to significant associations identified in our GWAS (Figure 6.6).

References

1. Sukumaran S, Xiang W, Bean SR, Pedersen JF, Kresovich S, Tuinstra MR, et al. Association Mapping for Grain Quality in a Diverse Sorghum Collection. The Plant Genome Journal. 2012;5:126.
2. Elshire RJ, Glaubitz JC, Sun Q, Poland JA, Kawamoto K, Buckler ES, et al. A Robust, Simple Genotyping-by-Sequencing (GBS) Approach for High Diversity Species. PLoS ONE. 2011;6:e19379.
3. Murphy RL, Klein RR, Morishige DT, Brady JA, Rooney WL, Miller FR, et al. Coincident light and clock regulation of pseudoresponse regulator protein 37 (PRR37) controls photoperiodic flowering in sorghum. Proc. Natl. Acad. Sci. U.S.A. 2011;108:16469–74.
4. Morris GP, Ramu P, Deshpande SP, Hash CT, Shah T, Upadhyaya HD, et al. Population genomic and genome-wide association studies of agroclimatic traits in sorghum. Proc. Natl. Acad. Sci. U.S.A. 2013;110:453–8.

Table 6.1 Correlations between traits in Kansas panel[a] and South Carolina panel

	**2012**	**2013**	**2014**
**Protein**	0.13 (p = 0.02)	0.19 (p = 0.005)	0.18 (p = 0.007)
**Fat**	ns	ns	ns
**Starch**	0.13 (p = 0.03)	0.21 (p = 0.002)	0.30 (p = 3.941e^{-06})

[a]Mean of 2007 and 2008

ns = not significant

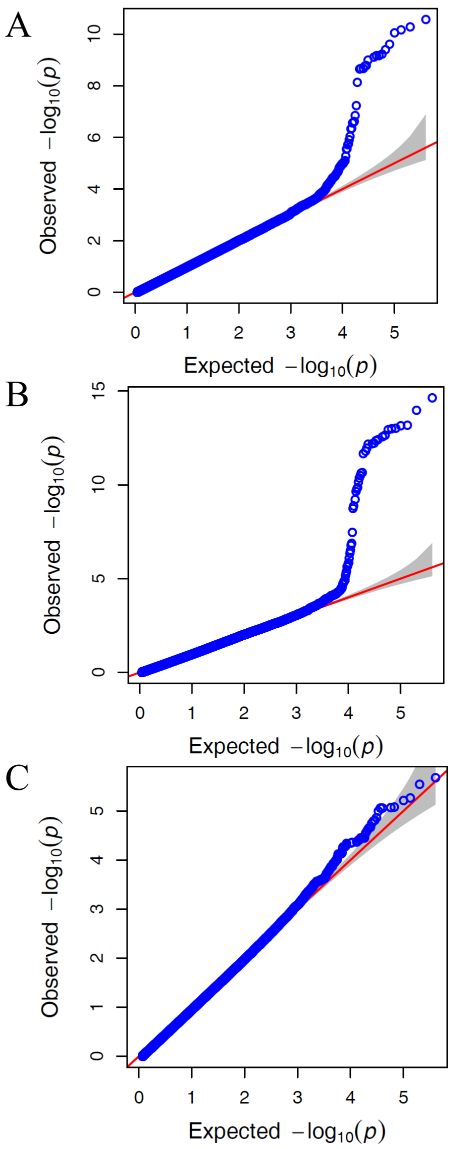

Figure 6.1. QQ plots of observed versus expected negative logarithms of the *P* values of SNPs from the MLM for A) protein, B) fat, and C) starch.

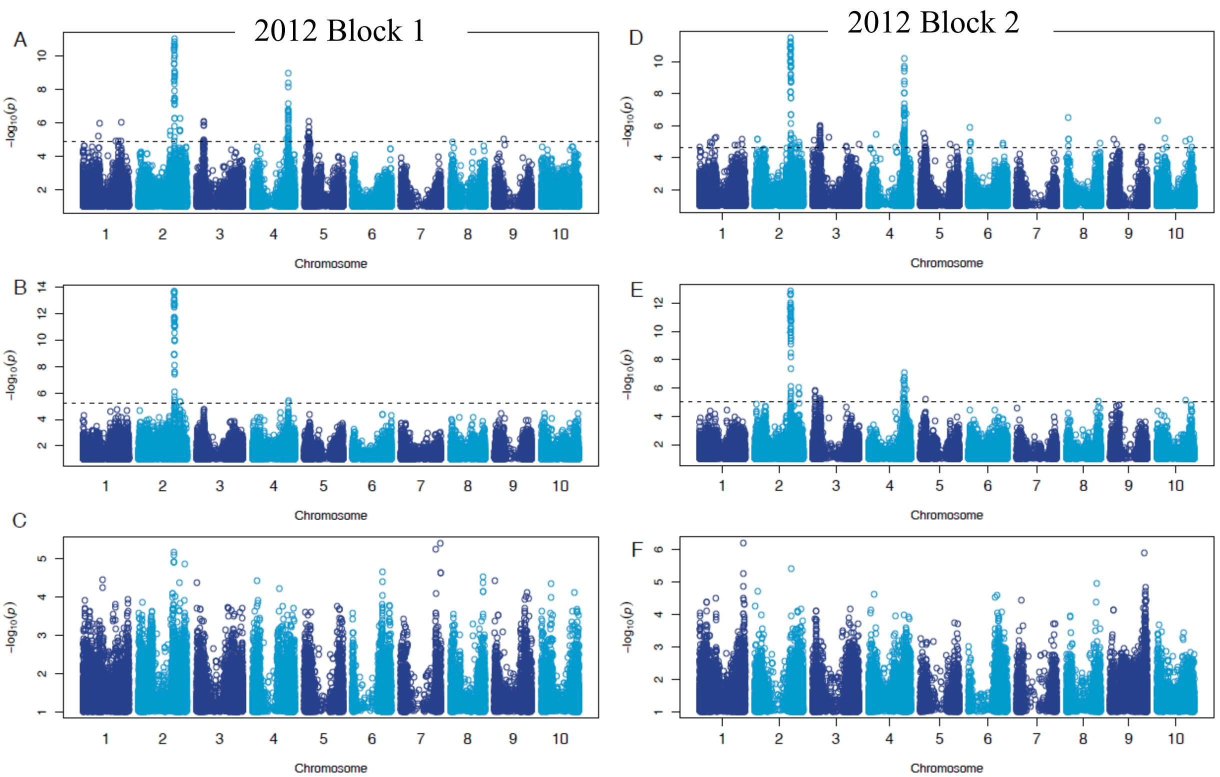

Figure 6.2. GWAS for protein, fat, and starch in two blocks grown in 2012. Manhattan plots of association results from a MLM analysis using 404,627 SNP markers and 218 accessions. Each point represents a SNP, with the -log10 p-values plotted against the position on each chromosome. SNPs with MAF < 0.05 were removed. The horizontal dashed line represents the genome-wide significance threshold at 5% FDR. (A) protein replicate 1; (B) fat replicate 1; (C) starch replicate 1; (D) protein replicate 2; (E) fat replicate 2; (F) starch replicate 2.

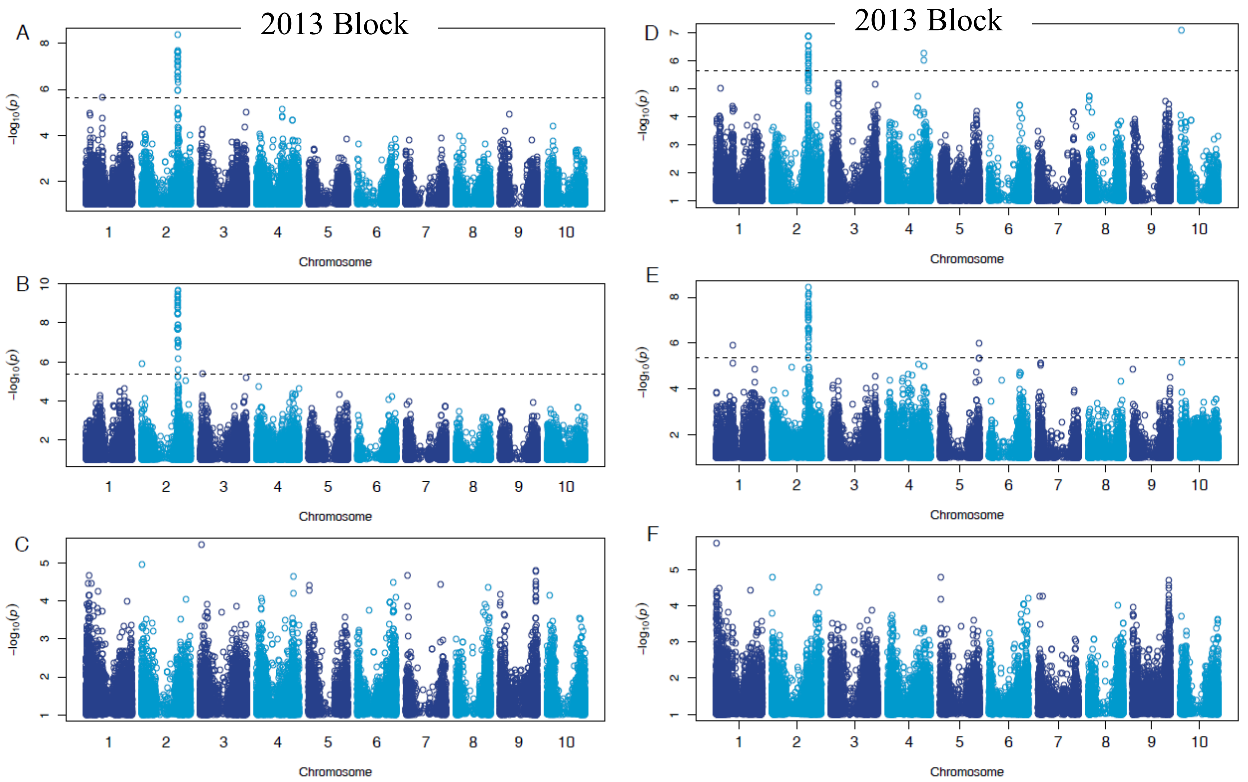

Figure 6.3. GWAS for protein, fat, and starch in two blocks grown in 2013. Manhattan plots of association results from a MLM analysis using 404,627 SNP markers and 277 accessions. Each point represents a SNP, with the -log10 p-values plotted against the position on each chromosome. SNPs with MAF < 0.05 were removed. The horizontal dashed line represents the genome-wide significance threshold at 5% FDR. (A) protein replicate 1; (B) fat replicate 1; (C) starch replicate 1; (D) protein replicate 2; (E) fat replicate 2; (F) starch replicate 2.

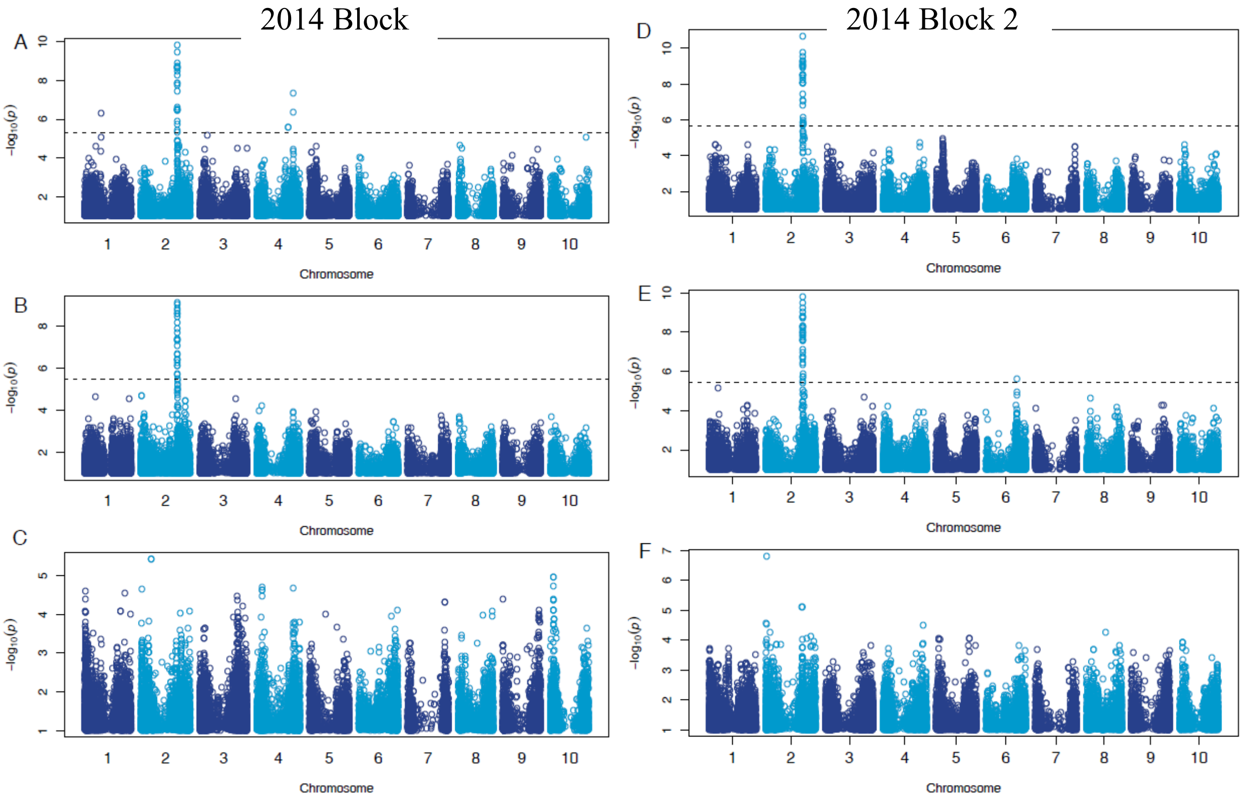

Figure 6.4. GWAS for protein, fat, and starch in two blocks grown in 2014. Manhattan plots of association results from a MLM analysis using 404,627 SNP markers and 300 accessions. Each point represents a SNP, with the -log10 p-values plotted against the position on each chromosome. SNPs with MAF < 0.05 were removed. The horizontal dashed line represents the genome-wide significance threshold at 5% FDR. (A) protein replicate 1; (B) fat replicate 1; (C) starch replicate 1; (D) protein replicate 2; (E) fat replicate 2; (F) starch replicate 2.

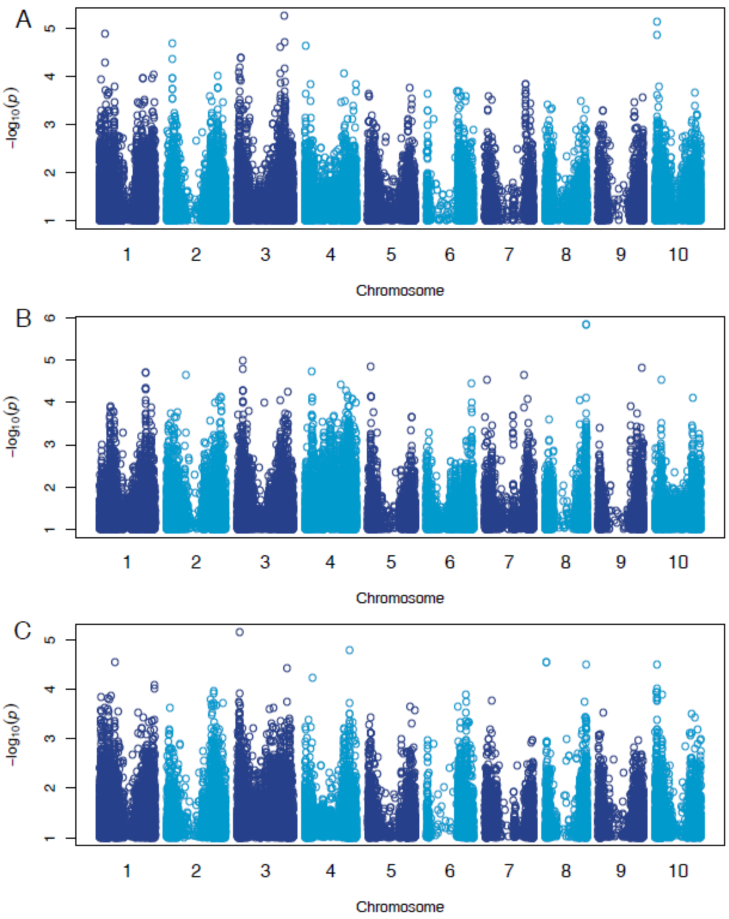

Figure 6.5. GWAS for protein, fat, and starch content in sorghum grain grown in Kansas. Manhattan plots of association results from a MLM analysis using 404,627 SNP markers and 239 accessions. Each point represents a SNP, with the -log10 p-values plotted against the position on each chromosome. SNPs with MAF < 0.05 were removed. (A) protein; (B) fat; (C) starch.

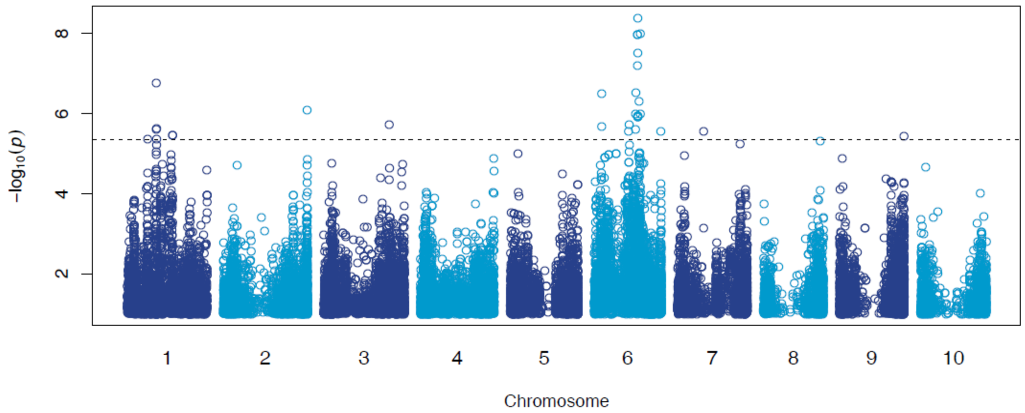

Figure 6.6. GWAS of flowering time in grain sorghum. Manhattan plot of association results from a MLM analysis using 404,627 SNP markers and 230 accessions. Each point represents a SNP, with the -log10 p-values plotted against the position on each chromosome. SNPs with MAF < 0.05 were removed. The peak on chromosome 6 around 42 Kb is near *ma1*.
